# The clinical significance of gut microbiota of chronic obstructive pulmonary disease with functional abdominal bloating and distension

**DOI:** 10.7717/peerj.20526

**Published:** 2025-12-18

**Authors:** Xiaowei Lu, Haiyun Dai, Xiang Gu, Jing Xie, Xianhong Zhong, Xiaofen Dong, Bing Su, Jia Su, Linlin Wang, Tingting Sun, Lihui Geng

**Affiliations:** 1 Jiangsu Province (Suqian) Hospital, Suqian, China; 2 Dinfectome Inc., Nanjing, China

**Keywords:** COPD, Gut microbiota, Biomarker, Diagnosis, NGS

## Abstract

**Background:**

Chronic obstructive pulmonary disease (COPD) is a disease with high morbidity and mortality. Functional abdominal bloating/distension (FABD), a functional gastrointestinal disorder characterized by recurrent sensations of abdominal fullness and/or visible abdominal distension without identifiable organic causes. FABD mainly impairs gastrointestinal functions—particularly intestinal transit and gas handling—rather than pulmonary function. This study characterized fecal microbiota in COPD patients with FABD to identify precision medicine biomarkers.

**Methods:**

Fecal samples from 20 COPD & FABD, 20 COPD, and 10 healthy controls (HC) were analyzed *via* metagenomic analysis. Gut microbiota diversity/composition were compared, and immune parameters (serum IgG, CD4+/CD8+ T cells) were assessed.

**Results:**

COPD/COPD & FABD patients showed significantly higher fecal microbiota α-diversity (COPD *vs*. HC: Chao1, *P* = 0.12; ACE, *P* = 0.14; Shannon, *P* = 0.0016; Simpson, *P* = 0.0013; COPD & FABD *vs*. HC: Chao1, *P* = 0.031; ACE, *P* = 0.031; Shannon, *P* = 0.00032; Simpson, *P* = 0.0005) *vs*. HC. β-Diversity analyses (PCA/PCoA) revealed distinct clustering between patients and HC (PCA, *P* = 0.014; PCoA, *P* = 0.013), but no separation between COPD and COPD & FABD (*P* > 0.05). Linear discriminant analysis (LEfSe) identified 50 discriminative biomarkers: 41 enriched in HC (*Bacteroides uniformis*), five in COPD & FABD (Bacilli, *Enterococcus faecium*), and four in COPD (*Streptococcus parasanguinis*). Notably, *Enterococcus faecium* was highly abundant in patients (22.04–26.92%) but absent in HC, suggesting a potential association with the COPD-FABD condition. Random forest models showed moderate diagnostic accuracy for all microbes (AUC = 0.632) and strong performance for fungal biomarkers (*Clostridium fessum, Clostridioides difficile*; AUC = 0.856).

**Conclusion:**

Gut microbiota signatures, particularly *Enterococcus faecium* and fungal taxa, may serve as non-invasive biomarkers for COPD progression and FABD diagnosis, warranting clinical validation.

## Introduction

Chronic obstructive pulmonary disease (COPD) is a chronic inflammatory disorder characterized by progressive pulmonary function deterioration, irreversible airflow limitation, chronic pulmonary inflammation, and small airway remodeling (Halpin et al., 2019, 2021). The prevalence of COPD is high, and it causes approximately 3.2 million deaths annually (GBD 2015 Chronic Respiratory Disease Collaborators, 2015). It is the fourth leading cause of death globally and the seventh highest disease burden (World Health Organization, 2000; Mathers & Loncar, 2006). Recent studies have shown that the respiratory microbial diversity of COPD patients in stable stage (SD) is significantly higher than that in acute exacerbation stage (AE), and the decrease in the abundance of key symbiotic genera (such as *Neisseria* and *Fusobacterium*) is closely related to the deterioration of the disease (Choi et al., 2024). These findings highlight the central role of microbiome in the pathological process of COPD. However, most existing studies have focused on the respiratory microbiota, and the role of the gut microbiota in COPD and COPD exacerbations requires further clarification.

Abdominal distension and bloating represent a prevalent and clinically significant comorbidity in COPD populations. According to the Rome IV criteria, functional abdominal bloating and distention (FABD) can be diagnosed when the diagnostic criteria for irritable bowel syndrome, functional constipation, functional diarrhea, or postprandial distress syndrome are not met (Drossman, 2016). Studies have demonstrated that altered gut microbiota in FABD patients may underline increased intestinal gas production or reduced gas clearance (Noh & Lee, 2020). Notably, while COPD is frequently associated with comorbidities such as cardiovascular disease, osteoporosis, and metabolic syndrome, FABD remains understudied, affecting up to 50% of COPD patients and profound impact on quality of life (Mari et al., 2019). Unlike systemic comorbidities, FABD might be associated with gut dysbiosis, offering a unique opportunity to explore microbiota-mediated mechanisms in COPD progression. This contrasts with other COPD complications, where immune dysfunction or systemic inflammation are more dominant pathways. Early diagnosis and precise treatment of COPD complicated with FABD are challenging. Traditional methods like fecal culture are time-consuming and have low positive rates (Blake et al., 2021). Therefore, it is necessary to develop a scoring system and identify specific biomarkers for these patients.

The gut microbiota has emerged as a research hotspot in COPD, with growing evidence highlighting its role in disease progression *via* the “gut-lung axis” to modulate immune regulation. For instance, germ-free mice exhibit profoundly reduced serum IgG levels and impaired immune responses upon pathogen infection (Tamaddon et al., 2025), while in diseases like diabetes and autoimmune disorders, the immune system acts as a critical bridge linking gut microbiota to host physiology (Guvenc & Danska, 2025). This concept extends to oncology, where a “gut microbiota–immune system–tumor” axis has been identified in colorectal cancer pathogenesis and immunotherapy responses (Pleguezuelos-Manzano et al., 2020; Nie et al., 2019). Accumulating evidence indicates that the pathogenesis and progression of COPD are closely associated with alterations in the composition and function of the intestinal microbiota (Bowerman et al., 2020; Li et al., 2020, 2021; Lai et al., 2022). In COPD specifically, recent studies demonstrate close associations between disease development and alterations in gut microbiota composition/function: Chinese COPD patients with varying severity (GOLD I-II *vs*. III-IV) show distinct microbial profiles, including reduced butyric acid-producing bacteria (*Faecalibacterium*) in mild cases and enrichment of potentially pathogenic (*Clostridioides difficile*) and pro-inflammatory (*Ruminococcus spp*.) taxa (Li et al., 2023). Collectively, these findings position gut microbiota as a key mediator in COPD immunopathology. However, it should be noted that most of these studies remain correlational and have not yet established causality or directionality in the gut–lung axis.

Given the high prevalence of FABD and its potential link to gut dysbiosis, which may involve mechanisms beyond the typical immune-related pathways observed in other COPD comorbidities, this study aims to characterize fecal microbiota signatures in COPD & FABD *via* next-generation sequencing (NGS) to identify potential microbial biomarkers for non-invasive diagnosis. Unlike other COPD comorbidities (*e.g*., cardiovascular disease), where immune-driven pathways dominate, FABD pathogenesis is uniquely linked to gut dysbiosis. This study therefore focuses on microbiota-mediated mechanisms underlying abdominal symptoms—a poorly explored dimension in COPD research. We aim to establish an evaluation system for identifying specific biomarkers that connect COPD-associated FABD with microbial dysregulation. By focusing on FABD, which affects over 50% of COPD patients yet lacks targeted diagnostic tools, our findings aim to bridge the gap between gut microbiota dynamics and clinical symptom management. This work seeks not only to clarify the gut-lung axis in COPD progression but also to lay the foundation for microbiota-driven interventions to improve symptoms and prognosis in this underserved patient subgroup.

## Materials and Methods

### Study design and patients

This study was conducted at the Department of Respiratory and Critical Care Medicine, Jiangsu Province (Suqian) Hospital, Jiangsu, China, between 2022 and 2024. Eligible participants were prospectively enrolled from the outpatient clinic according to predefined diagnostic criteria.

The sample size was determined based on previous studies investigating gut microbiota differences in COPD and associated gastrointestinal comorbidities, as well as the feasibility of outpatient recruitment (Li et al., 2023). We conducted prospective screening of all patients who visited the outpatient department of Jiangsu Province (Suqian) Hospital. A total of 50 subjects were included and divided into three groups: (1) COPD & FABD Group: 20 cases of COPD with FABD; (2) COPD Group: 20 cases of COPD; (3) HC Group: 10 cases of healthy controls. All patients had a body mass index (BMI) of 18.5–24.9 kg/m^2^. To avoid the impact of diet on the gut microbiota, each subject was required to maintain a stable diet within 2 weeks. All patients had similar lifestyle and dietary habits. Some of the most common foods include rice, seafood, dim sum, vegetables. The Ethics Committee of Jiangsu Province (Suqian) Hospital approved this study (2022-KYSB-0013), and written informed consent was obtained from all patients and health controls before the first sample collection.

The datasets presented in this study can be found online at: https://ngdc.cncb.ac.cn/gsa/browse/CRA024674, with access number CRA024674.

### Inclusion criteria and exclusion criteria

#### Inclusion criteria

Meets the diagnostic criteria for COPD as revised by the Chronic Obstructive Pulmonary Disease Group of the Respiratory Disease Branch of the Chinese Medical Association (revised edition of 2021): (1) Chronic cough or sputum production, dyspnea, and a history of recurrent bacterial lower respiratory tract infections (LRTIs), with prior antibiotic use before enrollment recorded and considered; (2) Exposure history to COPD risk factors; (3) Post-bronchodilator lung function test showing FEV1/FVC < 70%.

Meets the diagnostic criteria for FABD as defined by the Rome IV criteria. (1) Repeatedly feeling bloated or significantly bloating at least 3 days per month over the past 3 months; (2) Symptoms present at least 6 months prior to diagnosis; (3) Insufficient criteria for diagnosing irritable bowel syndrome (IBS) or functional dyspepsia.

#### Exclusion criteria

1. Does not meet the diagnostic criteria for COPD (applicable only to COPD & FABD group and COPD group); 2. Suffers from bronchial asthma, bronchiectasis, tuberculosis, diffuse panbronchiolitis, heart failure, lung cancer, and pulmonary embolism, among other diseases; 3. Suffers from functional constipation, IBS, functional dyspepsia (applicable only to COPD & FABD group); 4. Suffers from gastrointestinal perforation, bleeding, diffuse peritonitis, intestinal obstruction, gastrointestinal tumors; 5. Abdominal distension caused by the use of non-invasive ventilators; 6. Use of drugs affecting intestinal flora (including antibiotics and probiotics) within the past 6 months (all participants included had not received antibiotics or probiotics prior to enrollment); 7. Has a history of previous intestinal surgery.

### Measurements and data collection

Demographic data, clinical symptoms, medical history, medication history, and classification of COPD and lung function grading were collected from COPD & FABD group, COPD group, and HC group. Demographic and clinical data, including COPD classification and lung function grading, were collected from COPD & FABD group, COPD group, and HC group. Participants completed a gastrointestinal symptom questionnaire and assessed bloating severity using a visual analog scale. Fecal samples were analyzed by metagenomic analysis to clarify the fecal microbial profiles of the patients in the COPD & FABD group and COPD group and compared with the control group to comprehensively evaluate the diversity, composition, and distribution of the gut microbiota.

### Statistical analysis

For alpha diversity analysis, microbial ecological metrics were employed to quantify species diversity, evenness, and richness within microbial communities, including the Chao1 richness estimator, Abundance-based Coverage Estimator (ACE), Shannon index, and Simpson index. Beta diversity was assessed using ordination techniques, specifically Principal Component Analysis (PCA) and Principal Coordinate Analysis (PCoA), to characterize compositional dissimilarities between samples. Microbial community profiles were visualized *via* stacked bar charts and heatmap analyses to illustrate relative abundance patterns and intergroup compositional differences. Linear discriminant analysis effect size (LEfSe) was utilized to identify discriminative microbial taxa driving group-specific differences, with significance assessed *via* linear discriminant analysis (LDA) scores. Key taxonomic biomarkers were further validated using random forest modeling, with diagnostic performance evaluated *via* receiver operating characteristic (ROC) curve analysis. We used the relative abundance of gut microbiota as input features, training the model to learn the distinct microbial patterns between the COPD & FABD and HC groups, and then evaluating the model’s performance. The key output was a list of species, ranked by their MeanDecreaseAccuracy and MeanDecreaseGini scores, which are the most discriminatory for identifying patients with both COPD and abdominal symptoms.

Statistical analyses were conducted using SPSS 30.0 (IBM Corp., Armonk, NY, USA) continuous data were summarized as median (interquartile range, IQR), group differences assessed *via* Kruskal-Wallis H test with *post hoc* Mann-Whitney U tests, and categorical data compared using chi-squared tests. Statistical significance was defined as *P* < 0.05.

## Result

### Comparison of clinical data among COPD, COPD and FABD, and HC groups

**Figure 1:**
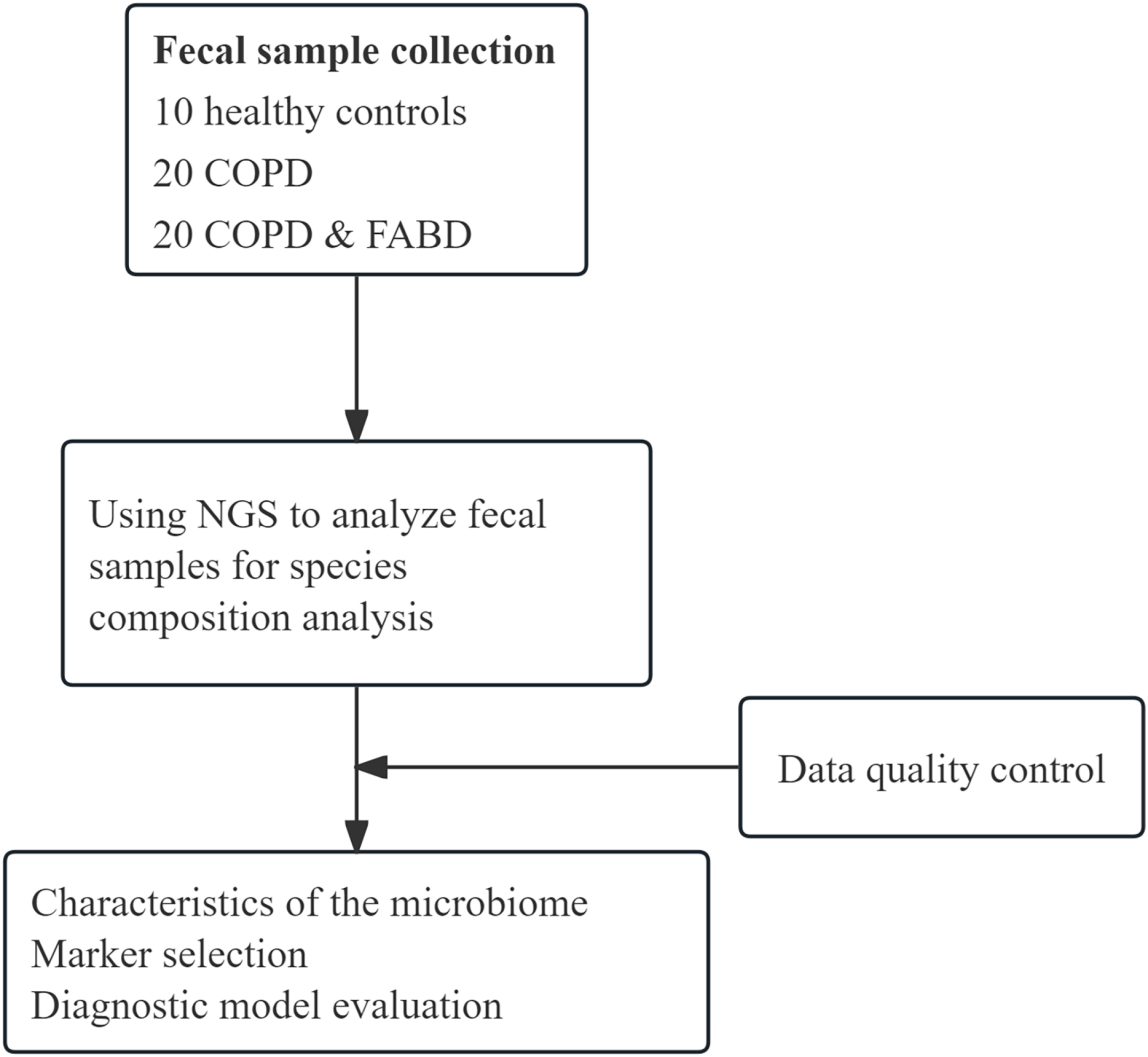
Schematic diagram of the study workflow. We collected 50 stool samples (20 from COPD patients, 20 from COPD patients with FABD, and 10 from healthy controls) for NGS testing for compositional diversity analysis of microbial flora.

We collected 50 fecal samples (20 from COPD patients, 20 from COPD patients with FABD, and 10 from healthy controls) for NGS testing (Fig. 1). The COPD group and the COPD & FABD group showed no significant differences in most laboratory and immunological indicators (Table 1). Furthermore, in the COPD & FABD group, there were 13 cases of mild abdominal distension, three cases of moderate abdominal distension, and four cases of severe abdominal distension.

**Table 1:** Patients’ baseline information.

Project	COPD (*n* = 20)	COPD & FABD (*n* = 20)	X^2^/Z	*P*
Age (years)	73.50 (69.25–79.00)	77.50 (71.50–79.75)	19.87	0.403
Gender [Count (%)]			4.286	0.362
Male	13	11		
Female	7	9		
Procalcitonin (ng/mL)	0.12 (0.08–0.47)	0.07 (0.05–0.10)	−1.837	0.066
GM test	0.15 (0.07–0.27)	0.14 (0.09–0.37)	−0.567	0.571
LWR	0.12 (0.06–0.17)	0.08 (0.04–0.18)	−1.02	0.307
NPR	0.038 ± 0.018	0.037 ± 0.015	−0.139	0.891
Alanine aminotransferase (U/L)	23.5 (17.2–29.9)	21 (14.4–30.750)	−0.642	0.521
Aspartate aminotransferase (U/L)	24.8 (17.3–33.5)	23 (18.15–28.3)	−0.529	0.597
Urea nitrogen (mmol/L)	6.3 (5.100–8.100)	7.3 (4.95–9.5)	−0.775	0.438
Creatinine (μmol/L)	58.87 ± 15.615	62.94 ± 19.044	0.656	0.517
Blood IgG	894.00 (734.00–1,070.00)	954.10 (714.90–1,067.15)	−0.038	0.970
C3 (mg/dl)	87.98 ± 28.258	82.90 ± 25.73	−0.532	0.598
C4 (mg/dl)	27.12 (22.13–34.47)	25.81 (17.59–33.94)	−0.359	0.720
CD4 + (cells/μl)	454.00 (238.00–584.00)	595.00 (198.50–1,063.50)	−0.926	0.355
CD8 + (cells/μl)	243.00 (174.00–524.00)	306.00 (76.50–491.50)	−0.340	0.734
Albumin (g/L)	37.00 (32.20–38.60)	32.00 (30.10–38.35)	−1.473	0.141

### Composition of the intestinal microbiota in the COPD, COPD and FABD, and HC groups

**Figure 2:**
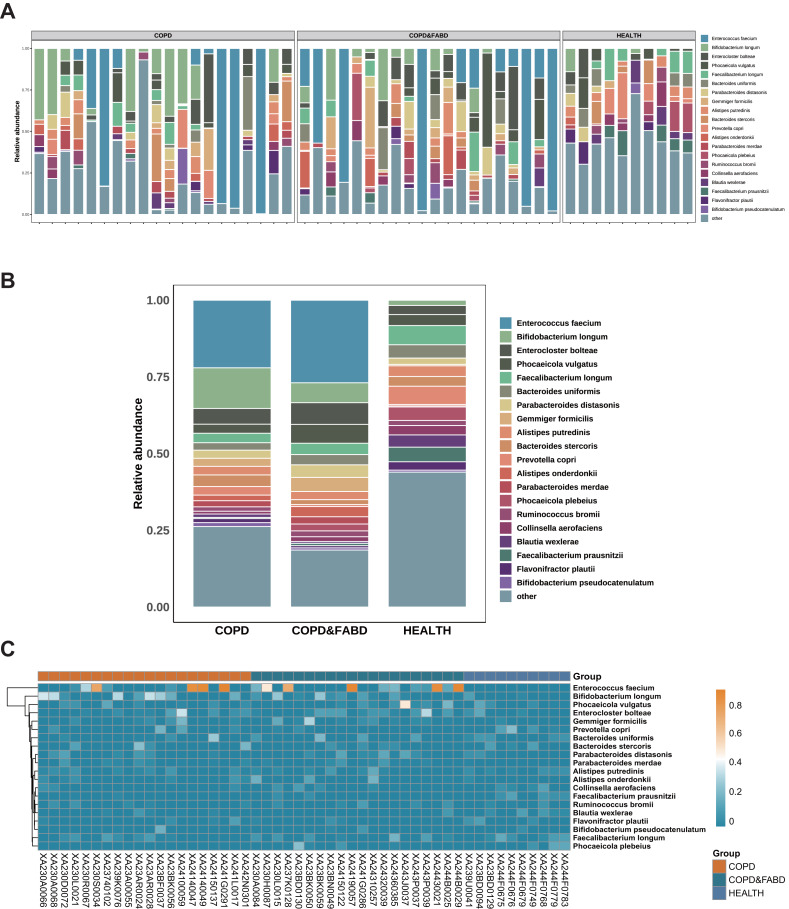
Analysis of microbial community composition in COPD and its comorbidities compared with healthy individuals. (A) Stacked bar chart of microbial community composition. (B) Comparative chart of relative abundance of microbial communities. (C) Heatmap analysis of microbial communities.

The top ten genera in relative abundance are *Enterococcus faecium*, *Bifidobacterium longum*, *Enterocloster bolteae*, *Phocaeicola vulgatus*, *Faecalibacterium longum*, *Bacteroides uniformis*, *Parabacteroides distasonis*, *Gemmiger formicilis*, *Alistipes putredinis*, and *Bacteroides stercoris* (Fig. 2A). In the COPD and COPD & FABD groups, *Enterococcus faecium* and *Bifidobacterium longum* are more abundant, with the COPD group at 22.04% and 13.24%, and the COPD & FABD group at 26.92% and 6.48%, compared to the HC group at 0% and 1.71% (Fig. 2B). The heatmap shows that the COPD and COPD & FABD groups have similar microbial phyla abundance, while the HC group has a distinct microbial community (Fig. 2C).

### Species $\alpha$ diversity and $ \beta$ diversity analysis

**Figure 3:**
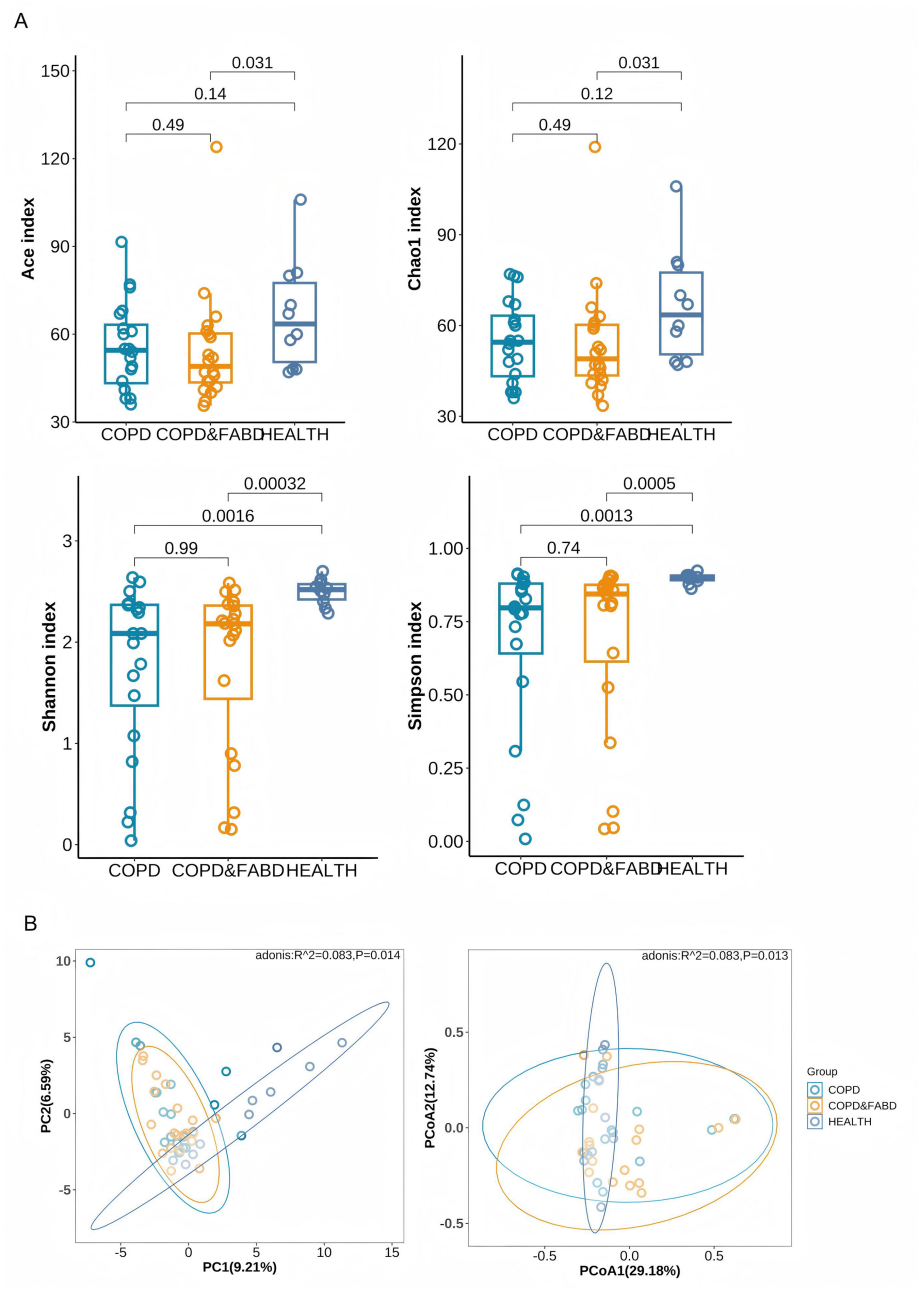
Analysis of microbial diversity in the COPD group, the COPD & FABD group, and the HC group. (A) Analysis of α diversity of species in the feces of the three groups of patients; (B) Analysis of β diversity of species in the feces of the three groups of patients.

The Chao1 and ACE indices showed that the COPD & FABD group had significantly lower richness than healthy individuals (Chao1, *P* = 0.031; ACE, *P* = 0.031). The Shannon and Simpson indices indicated that both the COPD and COPD & FABD groups had significantly higher richness and evenness than HC group (COPD *vs*. HC: Shannon, *P* = 0.0016; Simpson, *P* = 0.0013; COPD & FABD *vs*. HC: Shannon, *P* = 0.00032; Simpson, *P* = 0.0005), with no significant difference between the COPD and COPD & FABD groups (*P* = 0.99), (Fig. 3A). PCA and PCoA analyses revealed significant differences in fecal microbiota composition between the HC group and both the COPD and COPD & FABD groups (*P* < 0.01). Additionally, a significant difference was observed between the COPD and COPD & FABD groups (*P* < 0.05). These results indicate that while COPD and COPD & FABD groups are more similar to each other than to HC, compositional differences between these two patient groups remain significant (Fig. 3B).

### Species difference analysis

**Figure 4:**
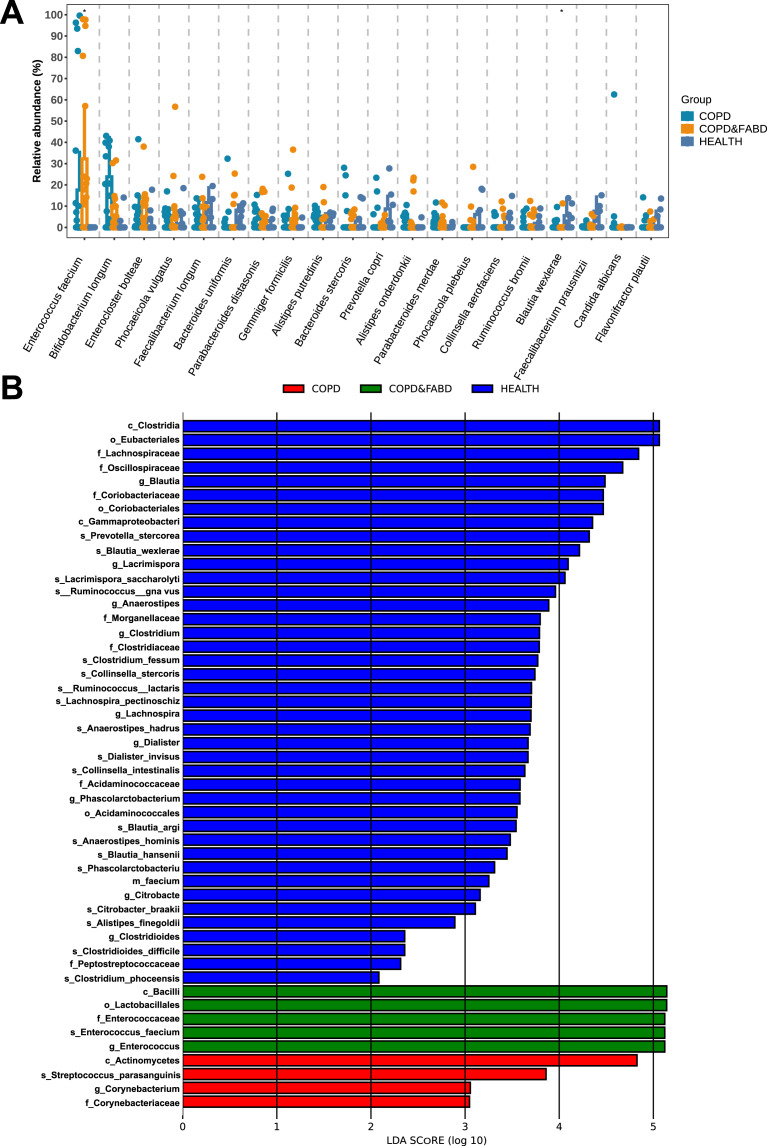
Analysis of microbial community structure and differences among different groups. (A) Comparison of the relative abundance of microbial genera within different groups; (B) Comparison of LDA scores of microbial genera between different groups. The figure shows species with an LDA Score greater than the set value of 2.0, where the length of the bar chart represents the impact size of the different species (*i.e*., the LDA Score), and different colors represent species from different groups. The x-axis represents LDA score values (>2), the y-axis represents the species name; the LDA score indicates the importance of the species to the grouping effect.

The relative abundance of microbial genera showed that the abundance of the top twenty species, including *Enterococcus faecium* and *Blautia wexlerae*, had significant differences among the three groups (*Enterococcus faecium*: *P* = 0.017; *Blautia wexlerae*: *P* = 0.015) (Fig. 4A). LEfSe analysis identified 50 discriminative biomarkers between the three groups, with four species in the COPD group, namely *Actinomycetes*, *Streptococcus parasanguinis*, *Corynebacterium*, *Corynebacteriaceae*; five species in the COPD & FABD group, including *Bacilli*, *Lactobacillales*, *Enterococcaceae*, *Enterococcus faecium*, *Enterococcus*; and 41 species in the HC group (Fig. 4B).

### Performance evaluation of key microbial biomarkers and diagnostic models

**Figure 5:**
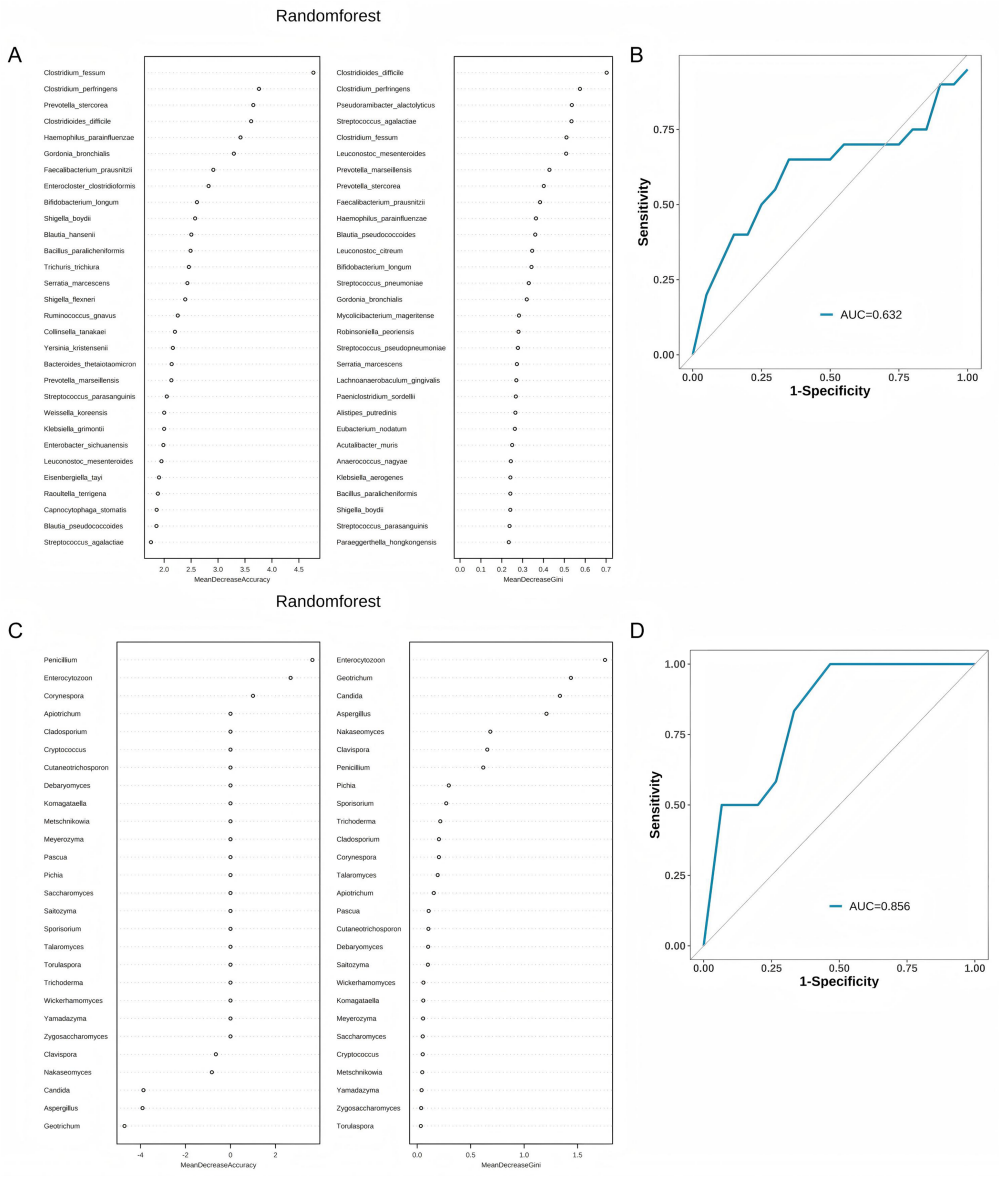
Performance evaluation of random forest analysis and classification models. (A) Importance analysis of all species in the random forest model; (B) ROC curves and AUC values of classification models for all species; (C) Importance analysis of fungal genera in the random forest model; (D) ROC curves and AUC values of fungal classification models.

Random Forest analysis demonstrated that *Clostridium fessum*, *Clostridioides difficile*, and *Clostridium perfringens* have higher importance (Fig. 5A). In the classification of all species, the area under the curve (AUC) of the Random Forest model is 0.632. This indicates that the model has a certain diagnostic capability (Fig. 5B). When we further analyze the model that only contains fungi (Fig. 5C), we find that the model performance has improved, and the AUC can be increased to 0.856 (Fig. 5D).

## Discussion

Currently, the gut microbiome’s utility in non-invasive diagnosis has been validated in colorectal cancer, hepatocellular carcinoma, and alcoholic liver disease (Yu et al., 2017; Ren et al., 2019; Smirnova et al., 2020), underscoring its translational potential across diseases. This study analyzed fecal microbiota profiles of healthy controls (HC), patients with COPD alone, and COPD comorbid FABD (COPD & FABD) *via* NGS. The results indicate FABD comorbidity induces subtle but specific gut microbiota adjustments on the basis of COPD-related dysbiosis, rather than completely reshaping the microbial pattern, which provides preliminary insights into the “gut-lung axis” mechanism underlying this comorbidity. While our cross-sectional study cannot definitively establish causal relationships, the observed microbial patterns, combined with insights from prior mechanistic studies, provide a framework for understanding the complex crosstalk between the gut microbiome and these comorbid conditions—highlighting the need for future longitudinal or interventional studies to validate directional causality.

Studies have shown that among the population aged ≥40 years that suffered COPD increase steadily to 90 million, indicating 13.5% prevalence (Zhong et al., 2007; Wang et al., 2018). In recent years, the potential role of gut microbiota in the pathogenesis of COPD has garnered increasing attention (Tamaddon et al., 2025; Kotlyarov, 2022). Previous studies have identified the presence of gut microbial dysbiosis in COPD patients and a plausible association between this dysbiosis and disease progression (Li et al., 2021). NGS has gradually become an important method for clinical diagnosis of difficult and unknown pathogens (Han et al., 2019), capable of detecting viruses, bacteria, fungi, parasites, and non-classical microorganisms (Simner, Miller & Carroll, 2018). Building on this foundation, the present study, with the aid of NGS testing for fecal pathogenesis, further revealed significant differences in the composition of fecal microflora between patients with COPD combined with FABD and healthy populations.

The impact of gut flora had been suggested to be associated with the progression of a variety of lung diseases, such as lung cancer, asthma, and pneumonia (Depner et al., 2020; Stevens et al., 2022). Emerging evidence suggests that gut microbiota influence COPD pathogenesis through multiple mechanisms, including modulation of systemic and pulmonary inflammation, immune cell homeostasis, and gut barrier integrity (Zhu et al., 2025). COPD is influenced by multiple environmental and host-related risk factors, among which cigarette smoking, air pollution, aging, and nutritional imbalance play predominant roles (Bowerman et al., 2020; Kotlyarov, 2022). Cigarette smoking, in particular, not only triggers airway inflammation and oxidative stress but also disrupts intestinal barrier integrity and alters gut microbial composition, thereby amplifying systemic inflammation through the gut–lung axis (Tamaddon et al., 2025; Wang et al., 2023). Moreover, dietary patterns characterized by low fiber intake and high fat content have been linked to reduced abundance of short-chain fatty acid (SCFA)-producing bacteria, such as Faecalibacterium and Roseburia, which may further impair mucosal immunity and lung homeostasis (Zheng et al., 2020). These findings suggest that COPD pathogenesis involves bidirectional crosstalk between the gut and the lung, in which shared environmental exposures and metabolic pathways drive chronic inflammation and microbial dysbiosis across both organs. Bowerman et al. (2020) first reported that poor lung function in COPD patients is linked to gut microbial changes, including a lower abundance of the family *Lachnospiraceae* and a higher abundance of *Streptococcus vestibularis*. Our observation of *Enterococcus faecium* enrichment (22.04% in COPD, 26.92% in COPD & FABD), which was absent in health controls. This discrepancy may reflect differences in disease stage. Zhu et al.’s (2025) cohort included more advanced COPD patients or the influence of FABD comorbidity, as *Enterococcus* is a known gas-producing genus directly linked to abdominal distension. In the present study, analysis of species composition revealed that the top ten genera in relative abundance, including *Enterococcus faecalis*, *Bifidobacterium longum*, and Clostridium baumannii, were largely shared between the COPD and COPD & FABD groups, indicating significant overlap in their gut microbial communities. However, distinct differences related to the presence of FABD were also observed and are further described below. Moreover, the abundance of microbial phylum was similar in the COPD and COPD & FABD groups, which was significantly different from that of the HC group. Although the overall microbial profiles between COPD and COPD & FABD groups showed high similarity, the LEfSe analysis identified five unique biomarkers in the COPD & FABD group included *Bacilli* and *Enterococcus faecium*, which may contribute to FABD-specific pathophysiology. For instance, *Enterococcus faecium* is a known gas-producing genus, and its higher relative abundance may be associated with the abdominal distension symptoms observed in FABD patients (Wei, Palacios Araya & Palmer, 2024). These findings suggest a potential link between COPD, FABD comorbidity, and alterations in specific gut taxa that could contribute to local gas accumulation or visceral hypersensitivity. However, the directionality and causality of these associations remain to be established, and further mechanistic studies are needed to clarify these relationships.

Emerging evidence demonstrates distinct microbiome dysbiosis along the oral-airway-gut axis in COPD patients compared to HC, with ecological perturbations exhibiting stage-dependent progression and severity-associated taxonomic shifts (Wang et al., 2023). In the analysis of species diversity, the Chao1 and ACE indices, as well as the Shannon and Simpson indices, revealed that the fecal microbiota of the COPD & FABD group is more abundant and diverse. The PCA and PCoA analysis showed that the COPD group and COPD & FABD group were significantly different compared with the HC group. This validates the uniqueness of gut microbiota in disease states. Notably, this elevation in α-diversity contrasts with studies reporting reduced diversity in advanced COPD (GOLD III-IV) (Drossman, 2016), suggesting that FABD comorbidity may introduce unique microbial signatures in early-to-moderate COPD. This difference may stem from two key factors: first, severe inflammation and frequent medication use in advanced COPD often reduce microbial diversity, whereas milder pathology in early-to-moderate COPD allows FABD-related microbial changes (*e.g*., increased gas-producing taxa) to elevate α-diversity. Second, FABD-specific microbial perturbations may introduce new components to the COPD gut community, further increasing α-diversity.

Beyond taxonomic shifts, the functional implications of dysbiosis in COPD-FABD require deeper exploration. For example, the elevated *Bacilli* in COPD & FABD (LDA > 3) are linked to Th17-mediated inflammation, which could exacerbate both pulmonary and intestinal barrier dysfunction (De Filippis et al., 2021). Additionally, the reduced *Faecalibacterium longum* (13.24% in COPD *vs*. 1.71% in HC)—a producer of anti-inflammatory butyrate, may impair mucosal immunity, potentially explaining the lower CD4+ T-cell counts in COPD patients (Kamlárová et al., 2025). This aligns with Thatrimontrichai, who observed reduced Faecalibacterium and serum butyrate levels in COPD patients, correlating with decreased CD4+/CD8+ T-cell ratios and systemic inflammation (Thatrimontrichai et al., 2025). The depletion of this key butyrate producer likely contributes to both intestinal barrier dysfunction and impaired lung immune surveillance *via* the gut-lung axis. These immune-microbiota interactions align with the observed serum IgG and complement level alterations, suggesting systemic immune-metabolic axis warranting mechanistic studies.

Performing further species difference analysis, we found that the abundance of *Blautia wexlerae* and *Enterococcus faecium* differed significantly among groups, with the latter dominating in both COPD (22.04%) and COPD & FABD (26.92%) but absent in healthy controls. Through LEfSe analysis, we identified 50 discriminatory biomarkers, including five unique to COPD & FABD included *Bacilli* and *Enterococcus faecium*—a genus linked to gas production, potentially explaining FABD-specific symptoms like abdominal distension. These findings align with emerging evidence that gut dysbiosis contributes to both respiratory and gastrointestinal pathologies (Ren et al., 2019; Zheng et al., 2020; Coker et al., 2022) yet uniquely highlight FABD as a comorbidity driven by microbial functional shifts rather than taxonomic overlap with COPD alone.

While our Random Forest model demonstrated moderate diagnostic utility for bacterial species (AUC = 0.632), this limitation likely stems from shared dysbiosis patterns between COPD and COPD & FABD groups, compounded by the small sample size. Notably, the fungal-specific model achieved higher accuracy (AUC = 0.856), suggesting mycobiome signatures included *Clostridium fessum* and *Clostridioides difficile* may better differentiate FABD comorbidity. This echoes recent advances in metagenomic biomarker discovery for digestive disorders but extends it to COPD-FABD—a population previously overlooked despite the high prevalence of abdominal symptoms (Thiele et al., 2024). To bridge this gap, our study not only mapped COPD-FABD microbial signatures but also proposed actionable targets gas-producing taxa for symptom-specific interventions. Future work should integrate multi-omics data (*e.g*., metabolomics of hydrogen/methane) and refine machine learning models to account for confounders like gender imbalance (45% female in COPD & FABD *vs*. 15% in COPD, *P* = 0.038) and unmeasured variables (*e.g*., smoking pack-years). Such efforts could unravel the causal “gut-lung” mechanisms underlying COPD progression and its heterogeneous comorbidities.

However, our study has several limitations. First, the COPD & FABD group had a significantly higher proportion of females (45% *vs*. 15% in the COPD-alone group, *P* = 0.038), and gender is a known modifier of gut microbiota composition (Pecyna et al., 2023). Although diet stability was controlled, other confounders such as BMI, smoking pack-years, and medications (*e.g*., inhaled corticosteroids) were not recorded, which may introduce bias into the observed microbial patterns. It is important to note that inhaled corticosteroids (ICS), commonly prescribed in COPD management, may influence both gut and lung microbiomes. ICS treatment has been shown to selectively alter the microbiome and host transcriptome in the small airways of COPD patients, potentially affecting immune responses and microbial diversity. Additionally, ICS can impact the composition and diversity of the respiratory microbiome, although the direction and consistency of these effects vary across studies. These changes in microbiome composition may contribute to disease progression and treatment outcomes, underscoring the need for further research to elucidate the mechanisms underlying these interactions (Yu et al., 2017). Also, in addition to healthy subjects serving as normal controls in this study, we have excluded patients who used antibiotics in the past 6 months, ensuring that the impact of medication on the included samples is minimized. However, this study did not include patients with isolated FABD, which prevents us from fully distinguishing the independent effects of bloating and COPD on the gut microbiota. Additionally, the relatively small sample size may have affected the statistical validity and generalizability of the results. Furthermore, the study lacked long-term follow-up data to accurately assess the dynamic relationship between intestinal flora changes and disease progression. Future studies should incorporate stratified analyses by gender, detailed confounder adjustments, expand sample sizes, and establish long-term follow-up mechanisms to enhance statistical power, clarify dynamic associations, and strengthen the diagnostic utility of the findings.

## Conclusion

This study identifies distinct gut microbiota signatures in COPD patients with FABD, particularly *Enterococcus faecium* enrichment and fungal biomarkers. Paving the way for non-invasive diagnostics and microbiota-modulating therapies to improve symptom management and prognosis in this underserved patient population.

## Additional Information and Declarations

### Data Availability

The following information was supplied regarding data availability:

The datasets are available at CNCB: CRA024674.
